# Afternoon Session, March 3rd, 1880

**Published:** 1880-05

**Authors:** J. A. Robinson


### AFTERNOON SESSION, MARCH 3rd 1880.

(Continued from Page 185.)

Discussion of question continued. Question: "Professional Culture, Scientific and Esthetic."

*Dr. Taft*: The second part of this question, I take it, includes only the truly esthetic culture and the ethical principles by which we should be governed. There should be one addition, it seems to me, and that is in reference to the ethical conduct of professional men. It has been brought to my attention in this wise: I have noticed that persons will avail themselves of many other things entirely foreign to the profession for the purpose of securing business. A professional man should not resort to any artifice to secure him business. I have known a dentist to unite with a church and make the remark that he thought it would be a good field to work in. No man in the dental profession should do such a thing. Men often put themselves into society, and they will join organizations of various kinds merely on the ground that it will be an advantage to them in their business. All things of this kind I regard as illegitimate methods of securing business, and if men in this profession or in any profession would consider it in this light, I am sure it would not be done; such practices should everywhere be discouraged. The subject should receive more attention than it does. Reproach has been brought upon members of the profession by the means they have employed to increase their business, and what effects one of the members of the profession, effects the whole, hence it ought to be discountenanced.

*Dr. Harlan* agreed with *Dr. Taft* in his views, and added, that there need be no fear of lack of business if men would thoroughly fit themselves for their profession. He thought men would not have to resort to joining Church in order to increase their business, still, that should not keep men from joining Church.

*Dr. Taft*: I would not be understood as advising dentists not to connect themselves with some Church; I am in favor of it, and believe all should belong to some religious denomination, but it is doing these things to attain the end which I have specified that I consider reprehensible.

*Drs. Taylor, Leslie*, and others expressed opinions, agreeing with what *Drs. Taft* and *Harlan* had said.

*Dr. Taft* then raised another point in the question: viz, devotion to the profession. He said; Some men are entirely devoted to their profession, while others use it only in a business way, just as they would a commercial or any similar business. We should consider the question, how far do I pursue my profession merely for gain. The great point should be to meet so far as we can the ravages of disease, and to restore to health the dental organs that have been rendered useless. Many dentists are more occupied with outside business than with their profession, and it is questionable whether such a one ought to occupy such a position. I have found some dentists engaged in politics, some in speculation, and sometimes in other things entirely foreign to their professional calling, so the mind and attention is diverted. We should exercise more care in that respect, and, if we practice this profession having for its object truly and really what we profess, we ought to give it the best of our time and attention. Everything else should be made to occupy a secondary place, as compared with our profession. There are some things of course, that are essential to the profession, but all ordinary pursuits such as politics, society association and mercantile pursuits can hardly be mixed up with it, to advantage. If we become proficient in our profession, we shall need all the time we may have, to devote to study and investigation, in order that we can perform the best work and do justice to our patients.

The subject was further discussed by *Dr. Jay* and others who concurred in the opinions already expressed.

*Dr. Leslie* spoke at some length and coincided with what had already been said. He remarked that there was a broad field for improvement and study for the dental student, and that it would depend on the young men in the profession whether much progress was made in the future. He added that it was each one's duty to endeavor to advance in the profession and to raise the standard of the colleges.

The subject was then passed and the next subject taken up was: "Is neuralgia a functional affection, or does it involve a structural change of the nerve tissue."

*Dr. Taft*: I do not know whether there is a change of the nerve tissue or not, and if others know, they have not told us. My impression is that there may be in some cases a structural change, but I do not consider it a material element. We know that neuralgia may exist in great violence for a very brief period and then be arrested for a longer or shorter period; during the period of cessation of pain, I take it that the organ is performing the proper work, is proceeding in its normal way. That may be said to be an assumption, but he who says it is assumption, let him prove it. There may be some change in some part of the nerve structure and neuralgia occur, and sometimes after it has accutally occurred, pain will be felt. After a manifest change has taken place in the nerve tissue, true neuralgia does not exist. Neuralgia occurs in a variety of ways. One of which is by impingement upon a nerve. In such cases structural change cannot be supposed to exist, for when the pressure is removed from the nerve the pain ceases and does not again return. If there was pain caused by structural change, the pain would not cease until the change was repaired.

*Dr. Osmond*: I am inclined to the opinion that where there is extraordinary inflammation, a change, a structural change must be produced. We have cases where neuralgia has been entirely relieved and after a time reproduce itself again; then, there have been cases where a portion of the lower jaw has been removed before the neuralgia could be relieved. I think where severe cases have been relieved and reappear again, it shows that there must be a structural change. I recollect I had a case in this college years ago, where a negro woman came to me, she was suffering the most agonizing pain, as if all the teeth in her head were aching at one time, and I had long before that removed all the teeth she had in her mouth, I examined her mouth and discovered a small lump, what is called a nueroma, a small growth, I removed it and the patient was relieved. It is a difficult matter to understand satisfactorily. We have neuralgia produced from various causes, both sympathetic and miasmatic, and it is also caused by any contraction upon the nerves that will interfere with the circulation, this will cause pain. Then again, poisons may be introduced and cause neuralgia. I don't think the subject is as well understood as it ought to be.

*Dr. Rawls:* I have always held to the theory that there can be no effect without a cause and that we could have no material effect in material things without a material cause. The distinction made by most pathologists between most diseases is this: that some are known as anatomical pathological conditions, and others as chemical pathological conditions or as dynamic conditions. But, what do they mean by functional diseases? Do they mean to say that the functional disease has changed the functions? They state as a rule that a functional disturbance is a disturbance that cannot be traced to any cause, except that it be distant, causing a functional change without affecting the material of which the organs are composed. If we knew whether a neuralgia occured as the result of a lesion or as the result of other things, then I would know how to treat it. There are operations that cause a change from a normal to an abnormal condition, and where does the pain come from, unless we have a disturbance of the elements that circulate through the system, the elements that compose the nerve tissue? It is pressure, you may say, but it is caused by a disturbance of the circulating elements, and that change or disturbance might amount to a structural change.

*Dr. Taft:* The nerves have a certain work to perform in the body; certain things must be accomplished in the proper performance of the work. The nerves must be nourished, and in order that this be done they must receive the proper amount of pabulum, and if they fail in this, they fail to perform their work. Then the eliminating function is another; a nerve may have debris remaining in it, this will cause interruption of the natural function ; this will cause pain, for the proper function is arrested ; the circulating fluid might be a little changed and this might lead one to infer there was a structural change ; but there may be a change of the fluids of the body without a structural change ; but what we understand by a structural change is a change from normal condition of a solid or soft solid body to an abnormal condition. I can conceive that there may be an interruption of the functions without a structural change at all ; there may be an interruption either by the want of nutrition or by the retention of debris, or clogging of the parts, and yet no structural change exist. The subjects of nutrition and elimination we should thoroughly understand. Poisons that are introduced into the nerves, as mentioned by Dr. Osmond, often cause Neuralgia ; they may impair the nutrition directly, or it go into the pabulum, and this injurious substance will in this way arrest the nutritive function or impair it. The eliminating function may be defective and in this way impair the function by preventing the supply of necessary nutrition.

*Dr. Osmond;* Is not neuralgia caused by reflex action ?

*Dr. Rawls* : What is reflex action ?

*Dr. Osmond* : It is action thrown back ; it is action conveyed back to the nerves by means of the spinal chord. For instance : neuralgia of the stomach is caused often by an affection of the uterus. During the period of gestation there is a drain upon the system and as a matter of course there is a poverty of the elements that go to keep the nerves in proper order.

*Dr. Rawls* : I don't seem to get hold of the subject as I would like. It is presumed that we have materials of the body that are furnished with nutrition. Let us call it material substance. If you disturb a part of the body or function by any process, it is affected either by depriving it of substance or by introducing any substance into it, either will render it abnormal ; then we can have nothing else but a change. The substance is material and the change will necessarily be material.

In malarious districts we have neuralgia caused by the poisons, and we administer medicines to relieve it and to tone up the system. What do we use medicines for, but for the purpose of restoring the system to its natural vigor? If there is anything lacking in the system to supply the want, there must be a material change in the substance, because to restore anything is to leave some effect. There are no restorations without the effect is left in the system. If there is a change or restoration of the system, it has undergone a material change and the medicine or its effects remain in the body.

*Dr. Osmond*: I don't know about that; if we had all the medicine in us now that we have ever taken some of us would be good sized drug-shops. We take quinine and arsenic as tonics, and if they were not rapidly eliminated they would kill us. The medicines merely assist the system in resisting the disease and enable it do its work more effectively and in that way throw off the disease.

*Dr. Rawls*: The medicines having gone into the system form a part of the particles of the system. The substance remains in the system. These restoratives are never found by any chemical tests to be in the same shape as when taken, the substance having gone into the system and gone through a change, it goes into the tissue and remains there?

*Dr. Taft*: What do you mean by restoratives?

*Dr. Rawls*: Something will restore something that is lost.

*Dr. Taft*: Simply nutrition?

*Dr. Rawls*: No; nutrition is entirely different. I think nutrition, or the substance that goes in as nutrition, after it has accomplished its work passes off, but restoratives never do pass out of the body.

*Dr. Taft*: I should like to hear Dr. Rawls discuss the question, he has been talking about material changes and has dodged the question entirely. I want to know whether he thinks neuralgia is a functional affection or whether it involves a structural change of the nerve tissue. If he thinks there is a structural change, let him say so, and if he don't know, let him say so. That restoring expression is a very elastic one; it must have been made of rubber and never vulcanized. The effects of restoratives are just as palpable if the parts are restored by one means or by an other. In malarial districts or where there is a predisposition to neuralgia, if you remove the poisons or whatever may obstruct the functions the disease will be relieved and there will be no structural change. You may make a fine invisible dianamic structure that we cannot get hold of, but what we want is something we can reach, see and know. It is well known that unequal preasure upon nerves will produce neuralgic pains; it is known that poisons will produce similar pain; it is also well known that by relieving the impingment upon a nerve the pain ceases; it is well known that any clogging of the functions will produce neuralgia, but after experiments from time to time and examinations under a microscope of nerves that have been variously affected, no structural change has been observed, and in my opinion all these abnormal conditions may exist and yet no structural change in the nerve tissue.

*Dr. Kempton* thought the subject was not as well understood as it would be in the future. He found it difficult to understand how an organ could depart from its function without some cause, although there were many derangements, the cause of which had not been discovered.

*Dr. Rawls* thought there could be no material effect without a cause, and if there was a disturbance the cause must work through material substance, if the nerves were disturbed there would be a contact with the muscles and bone, and a material disturbance of the functious would necessarily be a structural disturbance or change.

The next subject taken up for discussion was: Is treatment of inflamed or sensitive dentine by escharotics in accordance with true physiology?

*Dr. Taylor*: I proposed that question because I did not believe it was the right way to treat sensitive dentine, and I wanted the views of the profession on the subject. I take the ground that we should treat sensitive dentine just as we would inflamation anywhere else. I think the profession has gone astray in using escharotics on the pulps of teeth. If the acrid conditions of the fluids of the mouth are neutralized till the proper functions are restored, and as a general thing, if you have taken away all irritants you have got rid of sensitive dentine. I don't believe the profession at large is in the habit of properly preparing the mouth before filling the teeth. If the functions are restored you will find little trouble with sensitive dentine. If by carefully treating the mouth we can get rid of sensitive dentine, what is the use then, of using escharotics at all?

*Dr. Osmond*: I have had patients come to me with aching teeth, and I have examined their mouths and found the tooth of which they complained perfectly sound, while a little ways off from that was a tooth with the pulp exposed, but it did not ache. I have treated the diseased tooth and applied a healthy mouth wash and I have often relieved patients in this way. I have used chloride of zinc, but I now use a preparation of phosphoric acid and relieve sensitive dentine. But of late I have been applying nitrate of silver occasionally to the dentine with good results.

*Dr. Taylor*: I would recommend that all dentists should advise their patients to clean the teeth at night; many persons only clean them in the morning, but that is not the best time, for the remains of the debris of the day before, is in the teeth and the injury is mostly done during the night the teeth not being clean.

*Dr. Rawls*: I think Dr. Osmond must be mistaken in making use of nitrate of silver. We cannot have an application of the nitrate of silver to the dentine even in the weakest solution without a change in the albumen compounds, this amounts to the same change as the use of escharotics would make; it would be the same as nitric acid; it simply destroys. I do not think it is pathologically proper to obtund sensitive dentine. The question is, can we afford to break down particles of the dentine and leave it as an irritant. You often see cases where, when you have made an application, trouble will arise which would not have occured if you had not made the application.

*Dr. Osmond*: In the use of nitrate of silver you find in two or three months after it has been applied the tooth will be hard and it will have the appearance of the teeth of old tobacco chewers, and I will say that I think tobacco chewing in many cases will preserve soft and sensitive dentine.

*Dr. Rawls*: It is simply from the exercise of the jaws; it develops and hardens the parts that are brought into action.

*Dr. Osmond*: What is the reason that chewing gum does not make the teeth of young ladies to become hard?

*Dr. Rawls*: I suppose it does to some extent.

*Chairman*: The question is: Is the treatment of inflamed or sensitive dentine by escharotics in accordance with true physiology.

*Dr. Taft*: In order to understand this subject fnlly, it would be well to know something about sensitive dentine and the various stages in which it is found and the influences that affect it. It is found in all degrees from that which is scarcely perceptible to the most severe. The condition is always modified by systemic condition. I object to the general use of escharotics. The ordinary agents such as chloride of zinc, nitrate of silver, chromic acid and all these escharotic salts when applied upon the tissue produce decompositon, and when applied to dentine must have an injurious effect. Have not used escharotics for ten years because other resources are so abundant; if the sensitiveness is merely superficial it may be removed by cutting. It may be done by the excavator or the engine. Systemic treatment is generally desirable in such cases together with the local treatment. There are many obtunding agents that do not destroy tissue and there are so many varieties of other agents that may be employed advantageously that no dentist should ever use escharotics. A solution of chloroform and gutta percha can be used on the sensitive part, then put in the filling. The resources are now so abundant, and are every day increasing, that it is not necessary, to even talk about using escharotics; they are a thing of the past and should go with the past and be totally laid aside. Dr. Osmond speaks of the use of tobacco in preserving sensitive dentine; it is simply a stimulant and gives exercise for the jaws. I do not consider the use of tobacco proper on this account.

*Dr. Taylor*: The best way would be to abolish the whole of it.

*Dr. Taft*: There are some instances that have attracted my attention to the danger of using these things, so I have discarded nitrate of silver, chromic acid, etc.; there may be some cases when they may be used, but the cases are rare.

On motion the meeting adjourned until Thursday morning.

THURSDAY MORNING, MARCH 4th, 1880.

The president called the house to order at 9.30 and the subject: "Can Thermal Changes effect Fillings injuriously?" was called up.

*Dr. J. Taft* opened the discussion by saying he hardly knew what view to take of the subject. Perhaps it was in the mind of the maker of the question that the expansion of the gold fillings would operate injuriously on the teeth. Was that it, or was it that thermal changes would cause the gold to become disintegrated? Can the expansion of a gold filling, caused by thermal changes, to which it is subjected in the mouth, operate any injury upon the teeth? There are three things that should be taken into consideration. First: what is the amount of expansion in gold entirely solidified? The speaker here gave the rate of expansion according to the different tables and concluded it was so small in ordinary gold fillings, that no injury could result; that the teeth would expand sufficiently to accommodate any expansion that might occur in the mouth caused by thermal changes, for there never is any extreme change in the mouth. One table gave the rate of expansion as one in 682, and the teeth would certainly accommodate that. The teeth would expand in the same proportion as the gold of a filling. Dr. Taft produced a wire of solid gold, which, he said, he had experimented upon and it verified the tables. He said: I subjected it to a temperature of 32 degrees, embedding it in ice, then it was raised to a boiling point; by this it was found that the expansion was one in 650 degrees. The wire is 5 inches long, and if there is only that much expansion in a temperature varying 180 degrees, how much will there be in gold that is subjected to from 30 to 100 degrees? The variations are less from 70 to 200 than above and below that, so that the scale of variations that occur in the mouth will produce but slight expansion. Gold is the best conductor for thermal change we have; this quality cannot be removed, but the difficulty may be met in other ways, so it does not prove to be an insuperable barrier in filling teeth.

*Dr. Taylor*: When called upon to prepare a question I found that a great deal of the ground had been gone over, and I then thought of how many of our best operations fail. I first asked with reference to the use of escharotics. Then, in looking over some of our most artistic operations, I have found failures; some will remain years, then, if examined, there will be found a rough surface, and if you pass over with the instrument you find that the filling seems to be pushed out beyond the surface of the tooth a little, but not enough to be perceptable to the naked eye. Why is it? Is it because the fillings were not thoroughly condensed?

I know there is very much more ice consumed now than formerly and perhaps that has something to do with it, and taking into consideration the amount of hot drinks that are used, it may be that that has something to do with the failure of many of the best operations.

*Dr. J. Taft*: This gold wire was unannealed, and another fact is that upon removing the heat after the experiment, the wire would return to its exact size, and it is now as soft as lead wire. Now, if the filling would expand, it would return to its original size and position again. I have found fillings protruding, but they have become displaced. I have found it occur on the outside as well as upon the masticating surface. I do not think that thermal changes injuriously affect the fillings.

*Dr. C. R. Taft*: I think that in ninety nine cases out of a hundred where the fillings protrude, it is because the anchorage has given way and the block has lost its position. I think the swedging down comes through the process of mastication.

*Dr. Rawls*: There is undoubtedly an expansion in all metallic substances from heat and there is no power known in dyanamics that will prevent that expansion or contraction. The difficulty may be somewhat obviated by our mode of filling teeth. I have always advised dental students in filling with amalgam or gold to put a little lamina of some preparation of gutta Percha in the cavity before filling.

*Dr. Osmond*: I agree with Dr. Rawls in advising the use of gutta percha. I am inclined to believe thermal changes will effect gold fillings by expansion and contraction; it is likely to crack the enamel of teeth. I do not say it will affect small fillings, but it is more liable to affect the large ones.

*Dr. Porre*: A number of our fillings fail because of the neglect of the dentist to prepare properly the margin of the cavity. I take it that if the margin is well cut down to the solid portion of the tooth the filling will be more permanent. I think the tooth expands sufficiently to accommodate the expansion of the gold filling.

I notice some fillings are left rough and not finished as they should be. If the margin is well beveled off and prepared the results will be better. My impression is the pain in very many cases arises in teeth because some portion of the inside has been bridged over and air is confined in them; when a hot drink is taken this air expands and produces pain.

*Dr. Berry*: I apprehend if the gold fillings expand and contract to the injury of the teeth, we should have had the subject settled long ago, and we should not be here spending time in discussing the question. I have a tooth that had been filled 40 years before I examined it, and the filling stands as firmly now as when put in. Dr. Berry here produced the tooth.

*Dr. Jay*: I agree with Dr. Porre as to preparing the edges of the cavities. In preparing cavities it is my rule to cut the edges well down and build it square up even and smooth.

*Dr. Arnold* did not think thermal changes would affect injuriously gold fillings.

After some other discussion and illustrations of it, the subject was passed. The subject next discussed was: "The Progress of Practical Dentistry, Operative and Mechanical."

*Dr. Jay* opened the discussion by saying that those who had been in the profession for 20 years ought to know considerable about the subject. It was the fault of the person if he did not, and it was because he did not keep himself posted in dental matters. The dentist who did not keep up his knowledge of the progress made should not practice, but ought to withdraw from the business. Twenty years ago there were but few dental journals published. In 1855 and 1857 there were only two, the American Journal of Dental Science and The Dental Register. Dr. Jay remarked the improvements that had been made since that time, and closed his remarks by saying he had hopes that in the coming 20 years there would be as great improvements as there had been in the past quarter of a century.

The meeting was turned into an informal speaking meeting and all members who desired, related matters in their own experience.

*Dr. Kempton*: I am young in the profession and know but little. When I first began to practice, I used the oxi-chloride and use it yet. My plan is now to mix the oxi-chloride and apply it to the cavity before filling and I have better success since I have adopted that plan. It is the best application I have found for sensitive dentine.

*Dr. Jay*: I have found that application to be very good. I also use a mixture of a little of the oxide of zinc and oil of gloves and carbolic acid in small quantities. I have not used creosote for years. I was advised to try Dr. Fletcher's dentine for capping a nerve and the oxi-phosphates and since I have tried these I have not used oxi-chloride at all.

*Dr. Porre*: There has been a vast improvement in dentistry in the last 15 years. I remember that in this hall 15 years ago I gave my method of extracting teeth which was without cutting around the tooth as was the practice generally. I was called a barbarian, but now there are very few dentists who take a lancet and cut around a tooth before extracting. Some time after I went to St. Louis to the meeting held there and the subject of treating gums came up. I spoke of how I had used chloride of zinc with advantage and I was hissed. I believe it is an escharotic and will kill a nerve, if applied directly to it; but in treating inflammed gums, where the membrane is puffed up and tender, it has an admirable effect.

*Dr. Taft* spoke of the many inventions that had been made for the use of the dentist, and of how much the profession had advanced; that escharotics were being abandoned; that arsenic that had been formerly used was not used at all, or but seldom. All these had served a purpose, but better things have come and they are now discarded. The instruments used in the dental profession were also greatly improved. The best engine now made, for certain purposes, was the Elliott engine, as it produced no jar or uneven motion. He found some dentists using instruments that were clumsy which he could not work with satisfactorily. A dentist needs the best quality of instruments, and each one should strive to get the best as far as it was in his power.

The meeting here adjourned to meet at two o'clock P. M.

---

The Society met at two o'clock, pursuant to adjournment.

Subject for discussion: The progress of Practical Dentistry, Operative and Mechanical.

The discussion was resumed. *Dr. Van Antwerp* spoke on the subject and was of opinion, that mechanical dentistry had not made much improvement. He did not think the discovery and invention of rubber plates an improvement to dentistry. The tendency now was to cheapness and that in his opinion was a retrograde movement.

*Dr. Taft*: I will ask Dr. Van Antwerp if where rubber plates have been worn the trouble has been on account of more or less absorption.

*Dr. Van Antwerp*: I don't think I ever saw a case where there was not considerable absorption.

*Dr. Taft* remarked that rubber ought to be put on the obsolete list.

*Dr. Osmond* thought the trouble was not so much in the rubber as in the fact that people are in the habit of wearing their teeth at night. The bad effect would be removed if dentists would urge upon their patients the necessity of removing their teeth at night.

*Dr. Rehwinkel*: That is it; put it down to that.

*Dr. Keely*: Dr. Osmond has hit the nail on the head. I think dentists should instruct their patients to take their teeth out at night.

*Dr. Rehwinkel*: When I advised this years ago, I was laughed at. I have found ill effects of wearing gold plates as well as of rubber. The plate should always be carefully cleaned and removed at night. Dentists should so instruct their patients.

*Dr. Cameron*: I am opposed to anything like rubber. I think if all the dentists had done what I did 12 years ago there would not be a rubber plate made or worn now. The rubber made now is not as solid and hard as it was formerly; it was better then, and did not affect the mouth so injuriously as it does now. I am of the opinion that there is much more of coloring matter put in the rubber now than formerly. I dont think wearing teeth at night makes any difference. I always advise my patients to keep their teeth in at night. I have seen mouths with gold plates almost as bad as rubber. I don't know why it is, but I must take issue with Dr. Rehwinkel as to taking teeth out at night. I don't think it affects the mouth more than if they are worn in the day time.

*Dr. Rehwinkel*: It affects the capillary circulation by the pressure. If you wore your boots every night all the time, your feet would become sore just as the mouth does from wearing the teeth at night. The mouth has no rest and the circulation of the mouth is arrested, and of course it will become sore and inflamed.

*Dr. Cameron*: I have seen inflamed mouths from wearing rubber, silver or platinum plates, and I have put in some cases a new gold plate on the inflamed mouth and it has got well. I generally have refused to take an impression till the mouth is well, but I have put plates in sore mouths and they certainly have improved.

*Dr. Hunter* spoke on the subject and said he rose as the champion of rubber; for by the invention of rubber plates thousands were enabled to have teeth that could not afford to pay for a gold plate. To be sure the introduction of rubber plates had opened the door for an influx of incompetent men into the profession, but the good it did certainly counteracts the evil.

*Dr. Ullery*: I have used rubber for 20 years and I have seen as much evil effects from gold, silver and celluloid as from rubber.

*Dr. Taft*: The introduction of rubber into the profession has worked injuriously, in my opinion; it has let into the profession a countless number of men who are totally unfit for the profession. They have gone through the country advising the people to have their teeth out and have extracted every tooth they could get hold of and have advised new teeth because they were so cheap; never once thinking or caring whether the teeth could be repaired and saved; they only wanted to sell their teeth. This ought to be stopped. There should be laws to prosecute them. I will everywhere and on every occasion raise my voice against all such material.

*Dr. Jay*; I think if the people who come to your offices are talked and reasoned with they will be prevailed on in almost every instance to get the best article. A dentist should use discreation and always advise for the best.

The Association then adjourned.

